# MiR-186 bidirectionally regulates cisplatin sensitivity of ovarian cancer cells via suppressing targets PIK3R3 and PTEN and upregulating APAF1 expression

**DOI:** 10.7150/jca.41135

**Published:** 2020-03-13

**Authors:** Ying Xiang, Ya-Jun Chen, Yun-Bo Yan, Yu Liu, Jiao Qiu, Rui-Qiao Tan, Qing Tian, Li Guan, Shuai-Shuai Niu, Hong-Wu Xin

**Affiliations:** 1 Department of Cell Biology and Genetics, School of Basic Medicine, Health Science Center, Yangtze University, Jingzhou, Hubei 434023, China.; 2 Laboratory of Oncology, Center for Molecular Medicine, School of Basic Medicine, Health Science Center, Yangtze University, 1 Nanhuan Road, Jingzhou, Hubei 434023, China; 3 The First School of Clinical Medicine, Health Science Center, Yangtze University, Nanhuan Road, Jingzhou, Hubei 434023, China.; 4 Department of Oncology, Jingzhou Central Hospital, The Second Clinical Medical College, Yangtze University, Jingzhou, Hubei 434023, China.; 5 Department of Molecular Biology and Biochemistry, School of Basic Medicine, Health Science Center, Yangtze University, Jingzhou, Hubei 434023, China.

**Keywords:** ovarian cancer, miR-186, cisplatin sensitivity, dose-dependent, bidirectional regulation

## Abstract

Ovarian cancer is a highly lethal malignancy in the female reproductive system. Platinum drugs, represented by cisplatin, are the first-line chemotherapeutic agents for treatment of various malignancies including ovarian cancer, but drug resistance leads to chemotherapy failure. MicroRNAs emerged as promising molecules in reversal of cisplatin resistance. MiR-186 was reported to be downregulated in the cisplatin-resistant ovarian cell lines and miR-186 expression increased cisplatin sensitivity. However, we found the bidirectional regulatory effects of miR-186 on cisplatin sensitivity for the first time that overexpression of miR-186 at low concentration increased the cisplatin sensitivity of ovarian cancer cells A2780/DDP, while high concentration of miR-186 decreased the cisplatin sensitivity. The survival assay in other types of cancer cell lines verified the bidirectional regulatory function of miR-186 on cisplatin sensitivity in dose and cell type dependent manners. MiR-186 suppressed the protein levels of PTEN and PIK3R3 dose-dependently, which are opposite regulatory molecules of the oncogenic AKT pathway. MiR-186 also enhanced the protein levels of apoptotic gene APAF1 dose-dependently. We proposed the final effects of PTEN and APAF1 outweighed PIK3R3 when miR-186 at low concentration so as to increase the cisplatin sensitivity of ovarian cancer cells, while the final effects of PIK3R3 outweighed PTEN and APAF1 when miR-186 at high concentration so as to decrease the cisplatin sensitivity. We concluded the outcome of regulation of these opposite functional molecules contributed to the bidirectional regulatory effects of miR-186 in ovarian cancer cisplatin sensitivity. It deserves more attentions when developing therapeutic strategies based on the bidirectional functional miRNAs.

## Introduction

Ovarian cancer is the third most common malignancy and the second highly lethal malignancy in the female reproductive system, globally, according to the latest global cancer status data (GLOBCAN2018) 1. Platinum drugs, represented by cisplatin, are the first-line chemotherapeutic agents for various malignancies including ovarian cancer 2. Although the majority of patients with ovarian cancer can achieve complete remission at the initial stage with platinum-based chemotherapy, about 80% of patients show drug resistance at the later stage 3. Drug resistance leads to the failure of chemotherapy as well as in other cancers.

AKT is the most important effector of PI3K signaling. Class I PI3Ks phosphorylates PI4, 5P2 to PIP3, thus activating AKT. Phosphatase and tensin homolog (PTEN) dephosphorylates PIP3 to PI4, 5P2, negatively regulating AKT pathway. Activated AKT is able to promote cell survival, proliferation, and cause changes of metabolic pathways via its multiple downstream targets such as GSK3, FOXO and mTORC1 4. Inhibition of PI3K/AKT was reported to increase cisplatin sensitivity of cisplatin-resistant breast cancer cells 5. Recently, the AKT pathway was confirmed to be activated in cisplatin resistant ovarian cancer 6.

MicroRNAs (miRNAs), non-coding RNAs with a length of 19-25nt, regulates gene expression post-transcriptionally via binding to the 3'-untranslated regions (UTR) of target mRNAs 7. MiRNAs are essential in physiological and pathological processes, including in regulating cisplatin sensitivity 8. MiR-186 was documented as an tumor suppressor miRNA in the majority of studies, such as in carcinomas of prostate 9, breast 10 and liver 11, while some reports verified miR-186 as an oncomir 12, 13. We confirmed that miR-186 was downregulated in the cisplatin-resistant ovarian cell lines SKOV3/DDP and A2780/DDP cells, which were concordant to the previous reports 14, 15. However, we firstly found the bidirectional regulatory effects of miR-186 on cisplatin sensitivity in ovarian cancer cells: low concentration of miR-186 increased the cisplatin sensitivity of ovarian cancer cells, while high concentration of miR-186 decreased the cisplatin sensitivity. The survival assay in other cancer cell lines revealed that miR-186 increased cisplatin sensitivity was dependent on its dose and cell types.

To investigate the mechanism of the bidirectional regulatory effects of miR-186 in ovarian cancer cisplatin sensitivity, multiple softwares of targets prediction were used to explore the potential targets. PTEN, PIK3R3 (phosphoinositide 3-kinase regulatory subunit 3) and APAF1 (apoptotic protease activating factor‑1), were predicated as potential targets of miR-186. The dual reporter luciferase assay showed miR-186 inhibited all of these UTRs directly, and western blotting assay showed overexpression of miR-186 decreased the protein levels of PTEN and PIK3R3, which were two opposite functional molecules of AKT pathway, but increased the protein levels of apoptotic gene APAF1 in a dose dependent manner. We concluded the outcome of regulation of these opposite functional molecules contributed to the bidirectional regulatory effects of miR-186 in ovarian cancer cisplatin sensitivity.

## Materials and Methods

### Cell culture and Transfection

All human cell lines including cisplatin-resistant ovarian cancer cells SKOV3/DDP and A2780/DDP, and their parental cells SKOV3 and A2780, non-small- cell lung cancer cells A549, colon cancer cells HCT116, hepatic carcinoma cells HepG2, breast cancer cells SKBR3, and human embryonic kidney (HEK) 293T cells were conserved in our laboratory. The HCT116 cells in MycCoy's 5A medium (Senrui, China) and the other cells in Dulbecco's modified Eagle's medium (Invitrogen, USA) with 10% fetal bovine serum and 1% penicillin/streptomycin, were cultured in a humid atmosphere containing 5% CO2 at 37 ℃. Lipofectamine 2000 (Invitrogen) was used for transfection with oligonucleotides miR-186 mimic or NC (Gene Pharma Company, Shanghai, China). The NC was negative control, targeting none of mRNAs.

### Cell viability assay

Non-transfected or transfected cells in 96-well plates were exposed to various concentrations of cisplatin (Meilun, China) for 48 hours, then MTT (Sigma, USA) was added. Cell viability was assessed by the absorbance of each well read by microplate reader (Thermo scientific) at the wavelength of 492 nm.

### Quantitative real-time PCR

Cells were harvested 48 hours post-transfection. TRIzol reagent (Invitrogen) was used to extract the total RNA. The procedure of complementary DNA (cDNA) synthesis and real-time PCR was seen in the previous publication 16. The sequences of primers (Sangon Biotech, China) were listed in Supplymentary**Table S1**. U6 was used as internal control.

### Dual reporter luciferase assay

Three 3'-UTR recombinant constructs were generated using dual luciferase plasmid system (a vector containing the Renilla and Firely luciferase gene), including Luc-APAF1-3'-UTR, Luc-PTEN-3'- UTR and Luc-PIK3R3-3'-UTR, each of which including the potential biding sequences to miR-186. The Luc-3'-UTR vector and miR-186 mimic or NC were co-transfected into HEK 293T cells, 24 hours later, luciferase activity was tested 16.

### Western blotting

Proteins were extracted from cells 48 hours post-transfection with miR-186 mimic or NC. Antibodies to PTEN (Multi Sciences, Hangzhou, China), PIK3R3 (Multi Sciences), APAF1 (Bioss, Bejing, China) or GAPDH (Multi Sciences) were used. The procedure was seen in the previous publication 16. GAPDH was used as internal control.

### Statistical analysis

All experiments were conducted at least three independent performances, and data were shown as the mean ± standard deviation. Differences between two samples were analyzed by the two-tailed Student's t-test. Statistical significance was accepted at *P*<0.05.

## Results

### Downregulation of miR-186 in cisplatin-resistant ovarian cells

**Figure 1:**
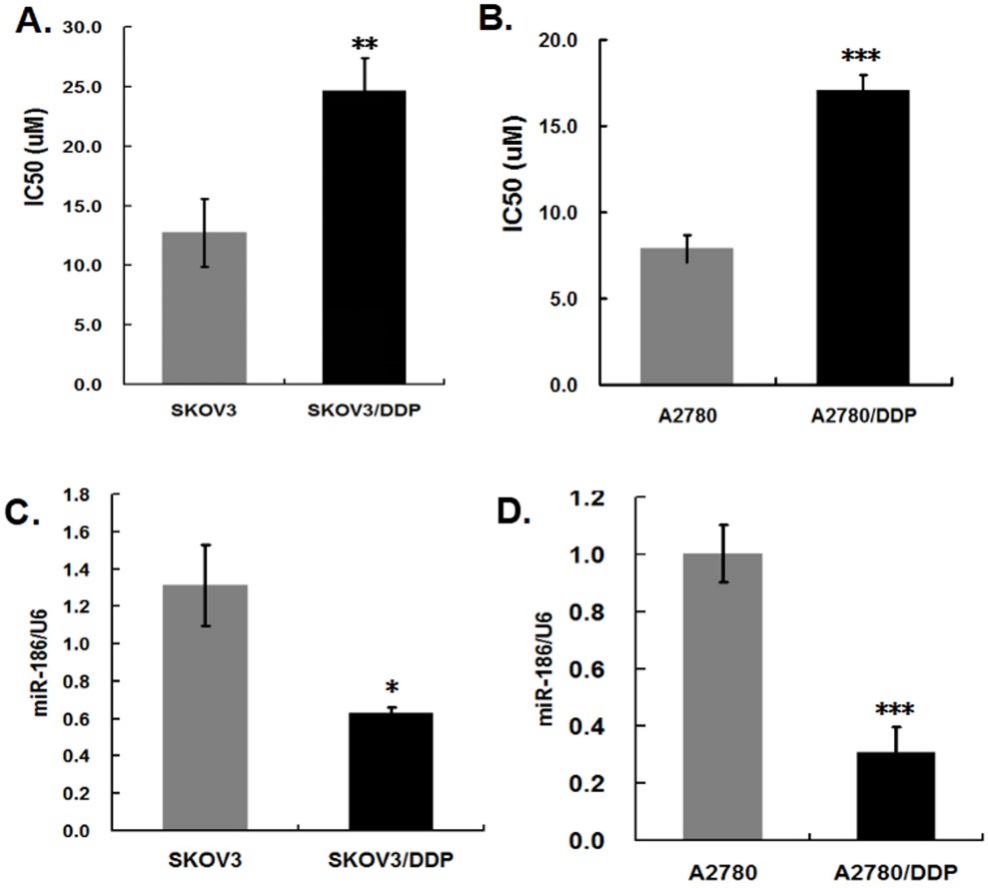
** The expression levels of miR-186 in cisplatin-resistant and the parental ovarian cancer cells. (A) (B)** Cells were treated with cisplatin for 48 hours, and then the IC50 of cisplatin was evaluated by cell viability assay. **(C) (D)** The expression levels of miR-186 was analyzed by quantitative real-time PCR in cisplatin-resistant and the parental ovarian cancer cells. *P<0.05, **P<0.01, ***P<0.001.

We confirmed the cisplatin resistance of SKOV3/DDP and A2780/DDP cell lines conserved in our lab. Cells in 96-well plates were treated with different concentrations of cisplatin, 48 hours later, the 50% inhibitory concentration (IC50) was assessed by cell viability assay. The IC50 of cisplatin was about two-fold in SKOV3/DDP and A2780/DDP cells than their parental SKOV3 and A2780 cells, respectively (Figure 1A, B), it revealed SKOV3/DDP and A2780/ DDP cells were indeed cisplatin-resistant ovarian cancer cells. To verify the alterations of miR-186 in cisplatin-resistant ovarian cells, total RNA was extracted from cisplatin-resistant ovarian cancer cells and their parental cells. The results of quantitative real-time PCR showed that the levels of miR-186 were markedly downregulated in cisplatin-resistant ovarian cancer cells SKOV3/DDP and A2780/DDP, in comparison with their corresponding parental cells (Figure 1C, D). Our results were concordant to the previous reports 14, 15.

### Bidirectional regulatory effects of miR-186 on cisplatin sensitivity of ovarian cancer cells

**Figure 2:**
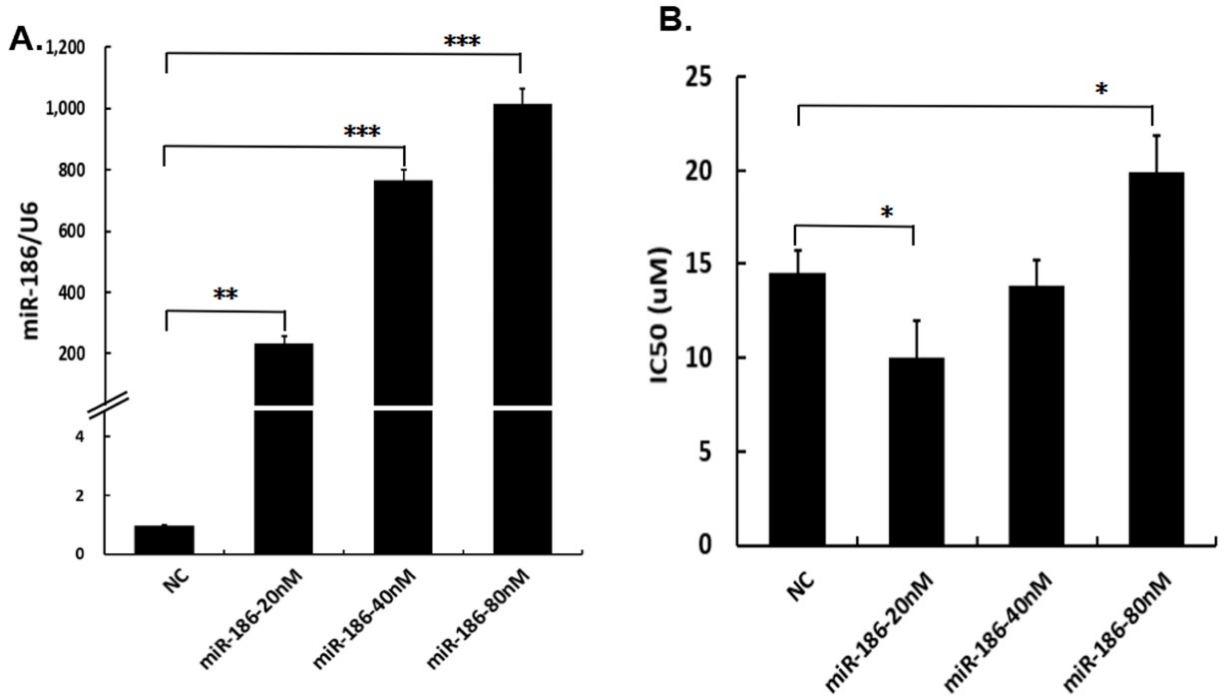
** The bidirectional regulatory effects of miR-186 on cisplatin sensitivity of ovarian cancer cells. (A)** A2780/DDP cells were transfected with different concentrations of miR-186 mimic or NC, 48 hours later, cells were collected and the levels of miR-186 were assessed by quantitative real-time PCR. **(B)** Transfected A2780/DDP cells with different concentrations of miR-186 mimic or NC, were treated with various concentrations of cisplatin for a further 48 hours, then the IC50 was evaluated by cell viability. *P<0.05, **P<0.01, ***P<0.001.

To study the role of miR-186 in regulation of cisplatin sensitivity, different concentrations of miR-186 mimic or NC were transfected into cisplatin-resistant ovarian cancer cells A2780/DDP. Total RNA was extracted 48 hours after transfection, and the transfection efficiency was evaluated by the expression levels of miR-186. The results of quantitative real-time PCR exhibited the levels of miR-186 were effectively increased by transfection of miR-186 mimic at 20nM, 40nM, 80nM, respectively (Figure 2A). The IC50 of cisplatin was assessed by cell viability assay. In comparison with the NC group, we found that the IC50 was downregulated in cells transfected with miR-186 mimic at 20nM, while the IC50 was upregulated when transfected with miR-186 mimic at 80nM and no significant change when transfected with miR-186 mimic at 40nM (Figure 2B). The results revealed that the treatment with miR-186 mimics at low concentration (20nM) increased cisplatin sensitivity of A2780/DDP cells but decreased cisplatin sensitivity at high concentration (80nM). It indicated miR-186 bidirectionally regulated cisplatin sensitivity of ovarian cancer cells in a dose-dependent manner.

### Bidirectional regulatory effects of miR-186 was dependent on cell types

**Figure 3:**
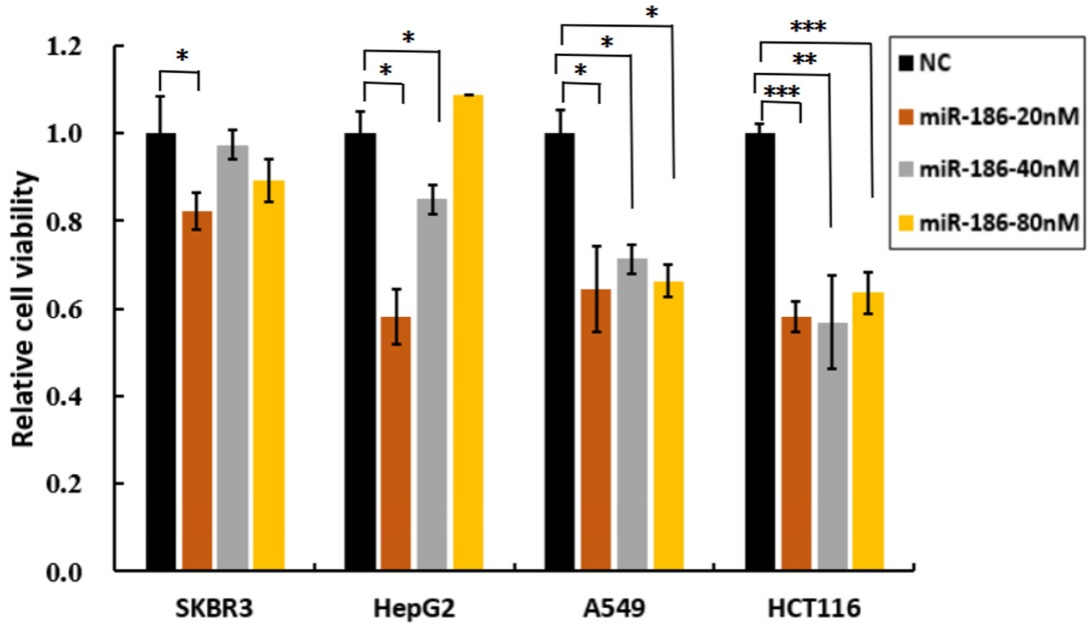
** The dose-dependent effects of miR-186 on cisplatin sensitivity in other types of cancer cells.** Transfected cells (including SKBR3, HepG2, A549 and HCT116 cells) with different concentrations of miR-186 mimic or NC, were exposed to cisplatin at the concentration of 20uM, and cell viability was analyzed 48 hours later. *P<0.05, **P<0.01, ***P<0.001.

To further investigate whether miR-186 play bidirectional regulatory effects on cisplatin sensitivity in other types cancer cell lines, SKBR3, HepG2, A549 and HCT116 cells were transfected with various concentrations of miR-186 mimic or NC, and then treated with or without cisplatin at 20uM. Cell viability assay showed transfection of miR-186 at the concentration of 20nM markedly inhibited cell survival in all of these cells compared with NC group, it revealed miR-186 mimic at the concentration of 20nM, increased cisplatin sensitivity in all of these cells. Obviously, miR-186 higher than 40nM lost its inhibitory effects in SKBR3, and miR-186 at 80nM lost its inhibitory effects in HepG2 cells. In A549 and HCT116 cells, miR-186 was effective in inhibiting cell viability, increasing cisplatin sensitivity even the concentration was as high as 80nM (Figure 3). These results revealed that the role of miR-186 in increasing cisplatin sensitivity was dependent on its dose and cell types. This may be due to various abundance of miR-186 and its targets in different types of cancer cells.

### The multiple opposite functional targets may contribute to the bidirectional regulatory effects of miR-186

**Figure 4:**
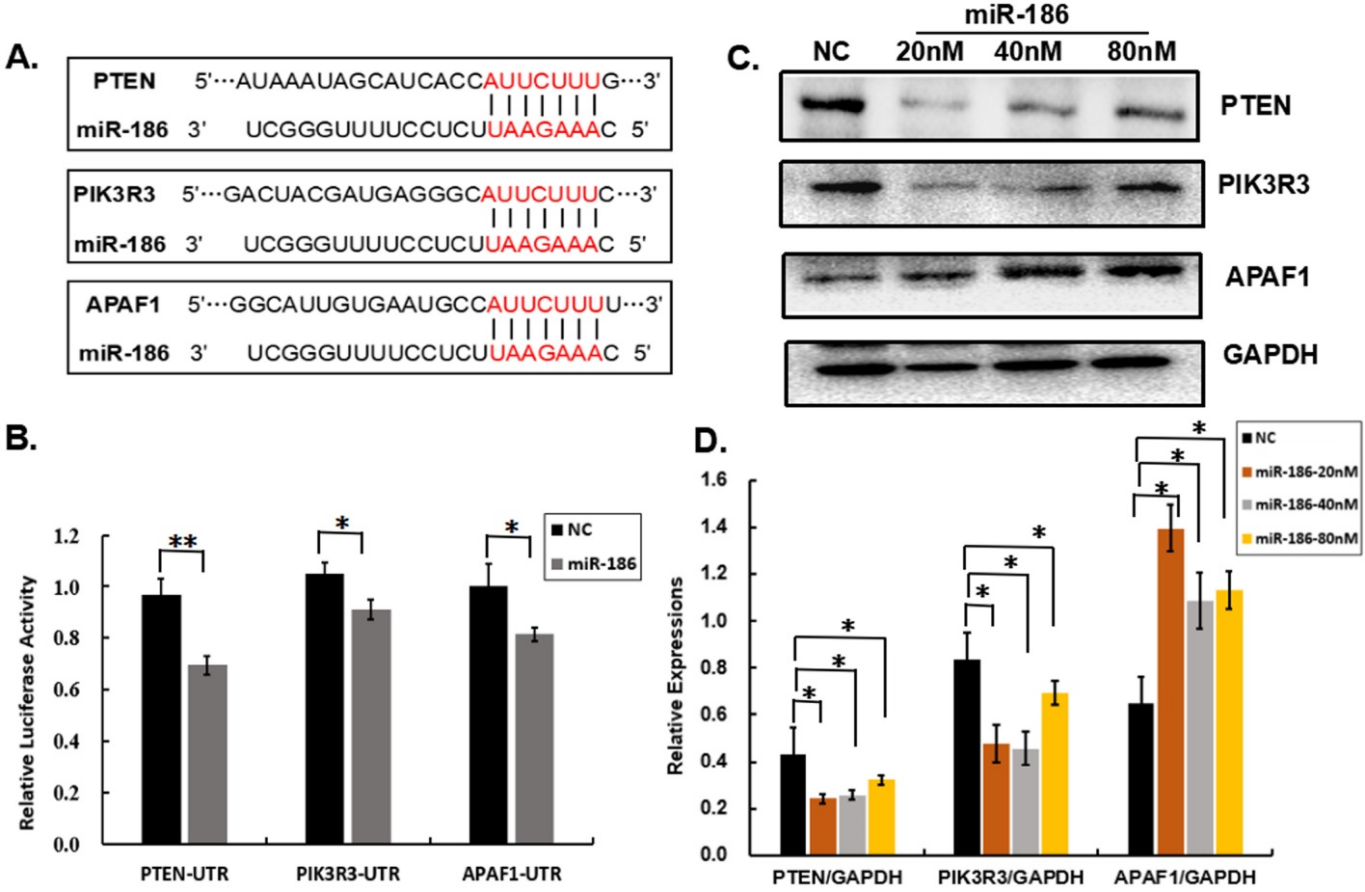
** Multiple targets of miR-186. (A)** The predicated targets PIK3R3, PTEN and APAF1 were showed in bioinformatics database. **(B)** HEK 293T cells were co-transfected with miR-186mimic or NC and a luciferase recombinant with the 3'-UTR of PIK3R3, or APAF1, or PTEN, then the relative luciferase activity was detected 24 hours later. **(C)** The protein levels of PIK3R3, APAF1 and PTEN were measured by western blotting 48 hours after transfection with miR-186 mimic or NC in A2780/DDP cells. **(D)** The quantitative analysis of protein levels (normalized to GAPDH) by Image J. *P<0.05.

In order to explore the mechanism of the bidirectional regulatory effects of miR-186 on cisplatin sensitivity of ovarian cancer cells, a series of bioinformatic softwares (including PITA, TargetScan, PicTar, microT, miRanda) were used to analyze the potential targets of miR-186 as far as possible. PIK3R3, PTEN and APAF1 were predicted to be potential targets of miR-186 (Figure 4A). PIK3R3, an important regulatory subunit of PI3K, promotes AKT activated. PTEN, a negative regulatory molecule of AKT pathway, is opposed to PI3K activity. Activated AKT is able to promote cell survival, proliferation. APAF1, an important molecule which combines with cytochrome c and Caspase9 precursor, leads to Caspase9 activation, thus promotes apoptosis 17. The dual-reporter luciferase assay showed miR-186 suppressed the 3'-UTR of PTEN, PIK3R3 and APAF1 directly (Figure 4B).

Overexpression of miR-186 significantly reduced the protein levels of PTEN and PIK3R3 in a dose- dependent manner compared with the NC group in A2780/DDP cells (Figure 4C, D). We concluded that PTEN and PIK3R3 were two targets in cisplatin-resistant ovarian cancer cells. However, miR-186 significantly increased the protein levels of APAF1 in a dose-dependent manner (Figure 4C, D). Moreover, we analyzed the co-expression of miR-186 and its targets in cancer tissues, and the data (collected from ENCORI database) showed an inverse correlation between miR-186 and PTEN, miR-186 and PIK3R3 in multiple cancer types, including in non-small-cell lung cancer, colon cancer, hepatic carcinoma, and breast cancer, and a positive correlation between miR-186 and APAF1 in several cancer types (Table 1). In addition, Dong *et al.* observed significant inverse correlation (r=-0.524, *P*<0.0001) between miR-186 and PIK3R3 in 200 EOC tissues 18. It validated that miR-186 could suppress the expression PTEN and PIK3R3, and enhance the expression APAF1.

**Figure 5:**
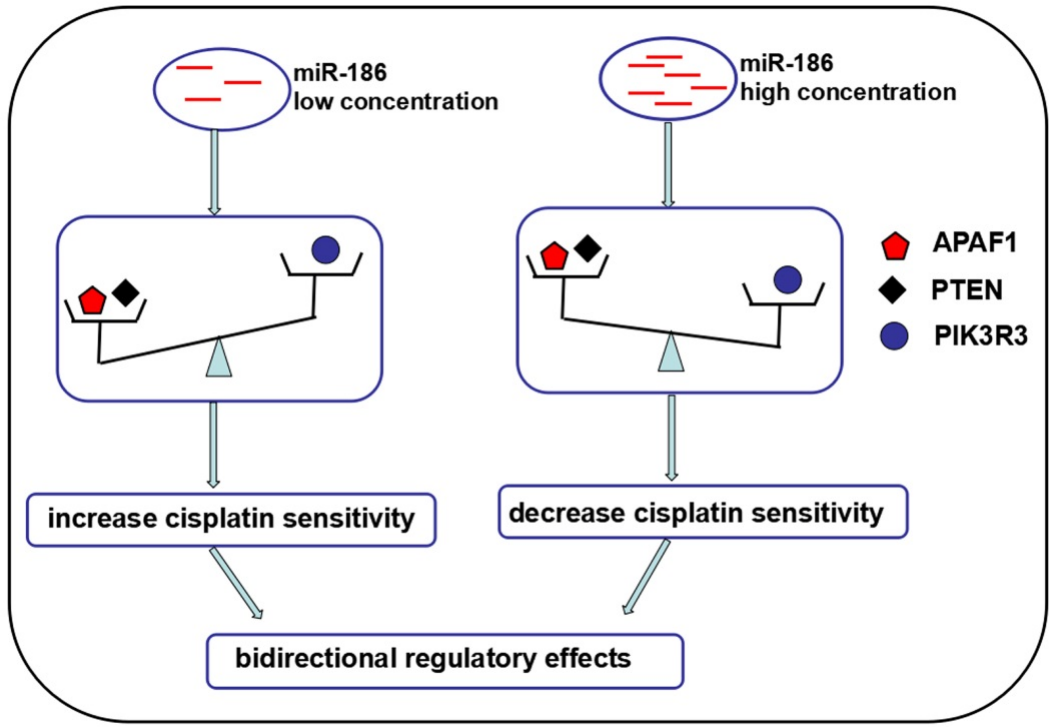
** The general view of multiple targets or regulated molecules contributing to the bidirectional regulatory effects of miR-186 on cisplatin sensitivity.** The final effects of PTEN and APAF1 outweighed PIK3R3 when miR-186 at low concentration so as to increase the cisplatin sensitivity of ovarian cancer cells, while the final effects of PIK3R3 outweighed PTEN and APAF1 when miR-186 at high concentration so as to decrease the cisplatin sensitivity.

Based on the above, we proposed the final effects of PTEN and APAF1 outweighed PIK3R3 when miR-186 at low concentration so as to increase the cisplatin sensitivity of ovarian cancer cells, while the final effects of PIK3R3 outweighed PTEN and APAF1 when miR-186 at high concentration so as to decrease the cisplatin sensitivity. We concluded the outcome of these opposite functional molecules contributed to the bidirectional regulatory effects of miR-186 on cisplatin sensitivity of ovarian cancer cells (Figure 5).

## Discussion

MiR-186, generated from the intron 8 of its host gene* ZRANB2*, was parallelly transcribed with its host gene 19. Majority of studies showed miR-186 served as a tumor repressor miRNA in various malignancies such as carcinomas of prostate 9, breast 10 and liver 11, which repressed proliferation and migration, and promoted apoptosis. Some studies verified that miR-186 served as an oncomir in endometrial cancer and squamous cell carcinoma, which promoted proliferation and migration, and inhibited apoptosis 12, 13 .

Zhu *et al.* collected series of ovarian cancer samples from patients with FIGO stage IIIC or IV (n=52), who were treated with the standard care of platinum-based therapy after surgery, and found miR-186 was greatly reduced in tumor specimens from patients with PFS (progression-free survival) <6 months (platinum resistant), compared with PFS>6 months (platinum sensitive) 15. MiR-186 was also downregulated in the cisplatin-resistant ovarian cell lines and ectopic overexpression of miR-186 increased cisplatin sensitivity *in vitro* 14, 15. However, we found the bidirectional regulatory effects of miR-186 on cisplatin sensitivity for the first time that overexpression of miR-186 at low concentration increased the cisplatin sensitivity of ovarian cancer cells A2780/DDP, while high concentration of miR-186 decreased the cisplatin sensitivity. Moreover, the bidirectional regulatory effects of miR-186 was also dependent on cell types (Figures 2, 3). The similarly bidirectional effects of another miRNA was also reported: miR-181a inhibited the cell viability of breast cancer cells significantly when miR-181a dose lower than 50nM, but promoted proliferation rather than inhibitory effect when miR-181a dose higher than 50nM 20. We concluded that miR-186 played bidirectional regulatory effects on cisplatin sensitivity in dose and cell type dependent manners.

We suspected multiple targets of miR-186 affecting pathways simultaneously contributed to its bidirectional regulatory effects. It was demonstrated that PTEN and PIK3R3 were two functional targets of miR-186 in lung adenocarcinoma 21 and in ovarian cancer 18. However, the abundance of predicated targets varies in different cell types 22. It is still necessary to verify whether they are functional targets of miR-186 in regulation of cisplatin sensitivity of ovarian cancer cells. We verified PIK3R3 and PTEN were the two targets of miR-186, and overexpression of miR-186 decreased the protein levels of PIK3R3 and PTEN in cisplatin-resistant ovarian cancer cells (Figures 4).

PTEN, a negative regulator of the PI3K/AKT signaling pathway, was an important molecule in regulating cisplatin sensitivity 23. Fu *et al.* showed both the mRNA and protein levels of PTEN was decreased in CDDP-resistant ovarian cancer tissues (N=5) compared with CDDP-sensitive ovarian cancer tissues (N=5) 24. Here were reports that PTEN was a direct target of miR-214 and miR-93 which induced cisplatin resistance in ovarian cancer 24, 25. That was to say miR-186 may decrease cisplatin sensitivity via suppressing PTEN. PIK3R3, one of the regulatory subunits of PI3K, could activate AKT pathway. In ovarian cancer, Zhang revealed PIK3R3 was upregulated significantly in cancer samples (N=28) compared with normal ovary (N=4) 26. Knockdown or silence of PIK3R3 decreased cell proliferation, migration and invasion, and increased apoptosis 27. Therefore, miR-186 may increase cisplatin sensitivity via suppressing PIK3R3.

APAF1, an important molecule promoting apoptosis 17, was downregulated in series of ovarian carcinoma samples with lymph node metastasis, and at the advanced FIGO stage 28. APAF1 was a validated target of miR-186 in cutaneous squamous cell carcinoma 12. In this study, the dual-reporter luciferase assay showed miR-186 suppressed the 3'-UTR of APAF1. However, overexpression of miR-186 significantly increased the protein levels of APAF1 in comparison with the NC group in A2780/DDP cells (Figures 4). We considered transfection of miR-186 mimic in A2780/DDP cells induced changes of targets pools or miRNA pools that lead to the upregulation of APAF1. Downregulation of APAF1 expression by miR-155 decreased the cisplatin sensitivity of A549 cells 29. Otherwise, upregulation of APAF1 gene expression contributed to miR-186 in increasing cisplatin sensitivity of ovarian cancer cells.

In conclusion, we verified that miR-186 was downregulated in cisplatin-resistant ovarian cancer cells, low concentration of miR-186 increased cisplatin sensitivity of ovarian cancer cells, while high concentration of miR-186 displayed the opposite function. The bidirectional regulatory effects of miR-186 was dependent on its dose and cell types. Further study revealed that miR-186 suppressed PTEN and PIK3R3 expression by targeting 3'UTRs directly, but increased the protein levels of APAF1. MiR-186 may increase cisplatin sensitivity by suppressing PIK3R3 and upregulation of APAF1, also may decrease cisplatin sensitivity by suppressing PTEN. We proposed the final effects of PTEN and APAF1 outweighed PIK3R3 when miR-186 at low concentration so as to increase the cisplatin sensitivity of ovarian cancer cells, while the final effects of PIK3R3 outweighed PTEN and APAF1 when miR-186 at high concentration so as to decrease the cisplatin sensitivity (Figure 5). We concluded the outcome of these opposite functional molecules contributed to the bidirectional regulatory effects of miR-186 in ovarian cancer cisplatin sensitivity.

## Supplementary Material

Supplementary table S1.

## Figures and Tables

**Table 1:** The Co-Expression Analysis for miR-186 and targets (from ENCORI database)

Correlation	Cancer Types	Sample Num.	Coefficient -r	p-value
**miR-186 VS PTEN**	Breast Invasive Carcinoma	1085	-0.172	1.17E-08
	Colon Adenocarcinoma	450	-0.112	1.80E-02
	Liver Hepatocellular Carcinoma	370	-0.11	3.42E-02
	Lung Adenocarcinoma	512	-0.161	2.64E-04
	Lung Squamous Cell Carcinoma	475	-0.096	3.68E-02
	Head and Neck Squamous Cell Carcinoma	497	-0.103	2.15E-02
	Kidney Renal Papillary Cell Carcinoma	289	-0.300	1.98E-07
	Bladder Urothelial Carcinoma	408	-0.227	3.71E-06
	Acute Myeloid Leukemia	83	-0.308	4.67E-03
	Brain Lower Grade Glioma	525	-0.093	3.36E-02
	Pheochromocytoma and Paraganglioma	183	-0.308	2.26E-05
	Rectum Adenocarcinoma	161	-0.302	9.82E-05
	Stomach Adenocarcinoma	372	-0.256	5.62E-07
	Uterine Corpus Endometrial Carcinoma	538	-0.145	7.17E-04
**miR-186 VS PIK3R3**	Testicular Germ Cell Tumors	156	-0.318	5.35E-05
	Thymoma	119	-0.305	7.31E-04
	Kidney Renal Clear Cell Carcinoma	517	-0.092	3.70E-02
**miR-186 VS APAF1**	Liver Hepatocellular Carcinoma	370	0.146	4.84E-03
	Esophageal Carcinoma	162	0.189	1.61E-02
	Kidney Renal Clear Cell Carcinoma	517	0.096	2.96E-02
	Skin Cutaneous Melanoma	449	0.242	2.10E-07
	Stomach Adenocarcinoma	372	0.111	3.17E-02

## Abbreviations

PTEN: phosphatase and tensin homolog
PIK3R3: phosphoinositide 3-kinase regulatory subunit 3
APAF1: apoptotic protease activating factor‑1
