# Supplementary material for: MiR-186 bidirectionally regulates cisplatin sensitivity of ovarian cancer cells via suppressing targets PIK3R3 and PTEN and upregulating APAF1 expression
Source: J Cancer. 2020 Mar 13;11(12):3446–53. doi: 10.7150/jca.41135 (PMC7150455; doi:10.7150/jca.41135)
Supplement: Supplementary file 1 — Supplementary table S1. [file jcav11p3446s1.pdf]

Table S1 The sequences of primers used in quantitative real-time PCR

Primers	Sequences
miR-186-sense	5’-CCGGCAAAGAATTCTCCT-3’
miR-186-anti-sense	5’-AGTGCGTGTCGTGGAGT-3’
U6-sense	5’-GCTTCGGCAGCACATATACTAAAAT-3’
U6-antisense	5’- CGCTTCACGAATTTGCGTGTCAT-3’
